# Exploring the associations of gut microbiota with inflammatory and the early hematoma expansion in intracerebral hemorrhage: from change to potential therapeutic objectives

**DOI:** 10.3389/fcimb.2025.1462562

**Published:** 2025-02-03

**Authors:** Haixiao Jiang, Wei Zeng, Fei Zhu, Xiaoli Zhang, Demao Cao, Aijun Peng, Hongsheng Wang

**Affiliations:** ^1^ Department of Neurosurgery, The Affiliated Hospital of Yangzhou University, Yangzhou, China; ^2^ Department of Neurosurgery, Yancheng First Hospital, Affiliated Hospital of Nanjing University Medical School, Yancheng, China; ^3^ Department of Medical Imaging, The Affiliated Hospital of Yangzhou University, Yangzhou, China

**Keywords:** gut microbiome, biomarker, brain-gut axis, hematoma expansion, intracerebral hemorrhage

## Abstract

**Background:**

Although a great deal of research has explored the possibility of a systemic inflammatory response and dysbiosis of the gut microbiota after an intracerebral hemorrhage (ICH), the relationships between gut microbiota and blood inflammatory indicators as well as their role in the hematoma expansion following an early-stage mild-to-moderate ICH (emICH) remain unknown. This study analyzes these changes and associations in order to predict and prevent hematoma expansion after emICH.

**Methods:**

The study included 100 participants, with 70 individuals diagnosed with emICH (30 with hematoma expansion and 40 without hematoma expansion, referred to as the HE and NE groups) and 30 healthy controls matched in terms of age and gender (HC). We used 16S rRNA gene sequencing to explore the gut microbial structure and its underlying associations with blood inflammatory parameters in the HE group.

**Results:**

Our findings showed a significant decrease in the diversity and even distribution of microorganisms in the HE group when compared to the HC and NE groups. The composition of the gut microbiota experienced notable alterations in the emICH group, especially in HE. These changes included a rise in the number of gram-negative pro-inflammatory bacteria and a decline in the level of probiotics. Furthermore, we observed strong positive connections between bacteria enriched in the HE group and levels of systemic inflammation. Several microbial biomarkers (e.g. *Escherichia_Shigella, Enterobacter*, and *Porphyromonas*) were revealed in disparateiating HE from HC and NE. Analysis of the Kyoto Encyclopedia of Genes and Genomes (KEGG) exposed disturbances in essential physiological pathways, especially those related to inflammation (such as the Toll-like receptor signaling pathway), in the HE group.

**Conclusions:**

Our exploration indicated that individuals with emICH, especially those with HE, demonstrate notably different host-microbe interactions when compared to healthy individuals. We deduced that emICH could rapidly trigger the dysbiosis of intestinal flora, and the disturbed microbiota could, in turn, exacerbate inflammatory response and increase the risk of hematoma expansion. Our comprehensive research revealed the potential of intestinal flora as a potent diagnostic tool, emphasizing its significance as a preventive target for HE.

## Introduction

Intracerebral hemorrhage (ICH) is the second most common cause of stroke (Lusk et al., 2023), which is associated with elevated mortality rates and leaving survivors with varying degrees of residual disability (Cordonnier et al., 2018; Li et al., 2021). Unlike ischemic stroke, medical and surgical interventions with demonstrable efficacy are scarce (Parry-Jones et al., 2020). Hematoma expansion, a key factor contributing to unfavorable outcomes, appears in roughly 30% of ICH cases, notably in the early phases (Yogendrakumar et al., 2021). Despite efforts to curb hematoma expansion, clinical trials have not greatly improved patient outcomes (Sprigg et al., 2018). Hence, rapid and precise identification of individuals who are prone to hematoma expansion and adverse outcomes via predictive tools is indispensable. Subsequently, administering anti-expansion therapies to positively identified patients holds promising clinical advantages.

To date, several studies have demonstrated that hematoma expansion could be closely linked to systemic inflammatory responses (Fonseca et al., 2021; Loan et al., 2022). For instance, Chu et al. (2023) revealed that the blood inflammatory parameters including C-reactive protein (CRP) and neutrophil-to-lymphocyte ratio (NLR) could be employed as predictors for hematoma expansion and ICH prognosis. Meanwhile, many studies have shown a strong connection between gut microbiota and systemic inflammatory response (Clemente et al., 2018). Gut microbiota, a dynamic ecosystem comprised of trillions of microorganisms, including bacteria, fungi, viruses, and archaea, resides in symbiotic harmony within the gastrointestinal tract (Armet et al., 2022; Sorboni et al., 2022). Beyond simple nutrition metabolism and digestion, the host and its resident microbes have a symbiotic relationship (He et al., 2022). It profoundly influences the development as well as the function of the host immune system, thereby exerting a decisive influence on systemic inflammatory responses (Tilg et al., 2020). On the other hand, it has been demonstrated that the gut microbiota could take part in the development of a number of neurological illnesses (such as Alzheimer’s disease (Xi et al., 2021) and stroke) via the gut-brain axis (Aburto and Cryan, 2024; Sorboni et al., 2022). For example, Stanley et al. (2018) pointed out that disturbances in the gut microbiota after stroke may lead to intestinal mucosal damage and inflammation, exacerbating the post-stroke neurological damage. Yin et al (Yin et al., 2015)discovered meaning dysbiosis in the gut microbiota of stroke patients, which was closely associated with clinical manifestations and disease progression. In addition, a growing amount of evidence suggests that the regulation of gut microbiota may offer innovative therapeutic approaches for mitigating stroke-related brain injury and promoting neurological recovery. For instance, Lian et al. (2023) discovered that improving intestinal microecology by supplementing with puerariae lobatae radix-resistant starch could be an effective therapy for stroke. Despite a multitude of studies exploring the connection between gut microbiota and stroke, most focus on ischemic stroke. There is limited research on the gut microbiota in the context of hemorrhagic stroke, especially in relation to early-stage mild-to-moderate intracerebral hemorrhage (emICH). Understanding the complex relationship between the gut microbiome and emICH could help identify potential new therapeutic approaches to prevent hematoma expansion. The current study was therefore designed to bridge this blank in the document.

In our research, we aimed to evaluate the influence of gut microbiota on the early expansion of emICH and its relevance with blood inflammatory parameters. By presenting novel therapeutic targets for the management of this disease, our study may contribute to the knowledge of the progression and treatment of emICH. The modulation of intestinal flora might be a promising underlying adjuvant therapeutic approach for the management of this disease in the future.

## Materials and methods

### Participants and study design

**Figure 1:**
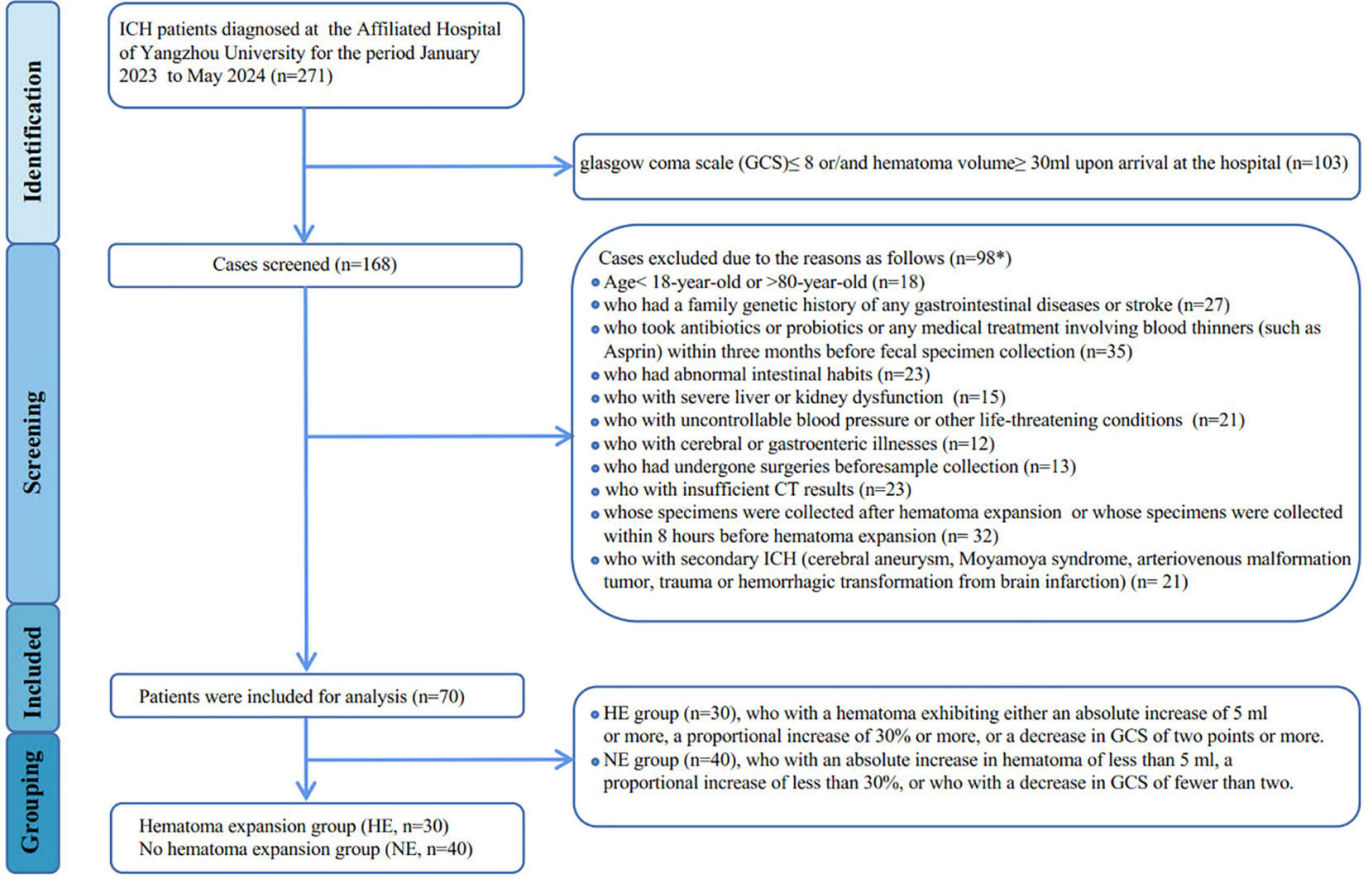
Flowchart showing derivation of study participants. *Patients may be excluded for more than one factor.

Between January 2023 and May 2024, patients with intracerebral hemorrhage at the Affiliated Hospital of Yangzhou University were diagnosed using computed tomography (CT) within 24 hours of the initial onset of symptoms, as assessed by the Department of Neurosurgery. Follow-up CT scans were conducted at 8 hours, 24 hours, and 48 hours after the initial scan to monitor hematoma expansion. Hematoma expansion is defined as either an absolute increase of 5 ml or more or a proportional increase of 30% or more in the hematoma on subsequent CT scans compared to the initial scan. Additionally, recognizing that the degree of consciousness disturbance due to hematoma expansion may vary based on the location of the cerebral hemorrhage, we also defined a decrease of 2 points or more in the Glasgow Coma Scale (GCS) as indicative of hematoma expansion. An analysis of bio-clinical and demographic data was performed for all participants, which included laboratory tests to assess blood inflammation levels, encompassing high-sensitivity C-reactive protein (hs-CRP), procalcitonin (PCT), interleukin-6 (IL-6), serum amyloid A protein (SAA), monocyte-to-lymphocyte ratio (MLR), and neutrophil-to-lymphocyte ratio (NLR). The exclusion criteria are detailed in the patient screening flowchart (**Figure 1**). Ultimately, 100 participants provided informed consent, consisting of 30 healthy controls (HC) and 70 newly diagnosed ICH patients (classified as mild-to-moderate, with a GCS>8 and hematoma volume <30 ml), which were further divided into two groups: the hematoma expansion group (HE, n=30) and the no hematoma expansion group (NE, n=40).

### The process of extracting DNA and collecting samples

The standardized procedure for the collection and extraction of genomic DNA was employed. Fresh fecal samples were procured within a 48-hour timeframe immediately upon admission to the hospital, using sterile cotton swabs. Subsequently, the samples were preserved without delay in a designated solution and were then stored at -80°C for additional analysis. We employed the PowerMax extraction kit to extract DNA, which was then preserved at -80°C to uphold its integrity. To determine the concentration and quality of the DNA, the NanoDrop ND-1000 spectrophotometer from Thermo Fisher Scientific was utilized. After collecting fecal samples, we immediately gather blood samples for blood routine and inflammatory indicator tests.

### 16S rRNA amplicon pyrosequencing

Using the specific primers (515F and 806R), we successfully amplified the bacterial 16S rRNA gene’s V4 region by PCR. For the purpose of multiplex sequencing, we strategically integrated sample-specific barcodes into the TruSeq adaptors. To optimize thermal cycling, an initial denaturation step at 98°C for 30 seconds was performed, laying the foundation for subsequent amplification. This preceded 25 cycles, each comprising a 15-second denaturation at 98°C, a 15-second annealing at 58°C, and a 15-second extension at 72°C. Following this, a final extension step of 1 minute at 72°C was carried out. Subsequently, the yield underwent purification with AMPure XP Beads from Omega Bio-Tek in the USA. To quantify the PCR amplification outcomes, we utilized the PicoGreen dsDNA Assay Kit sourced from Invitrogen in the USA. Subsequently, amplification products of equal quantities were pooled, and sequencing was conducted on the Illumina HiSeq4000 platform, adopting a paired-end 2×150 bp sequencing strategy.

### Sequencing data analysis

The analysis started with aligning the paired-end reads to their corresponding samples, facilitated by the use of unique barcodes. The barcodes were then removed along with primer sequences, thus simplifying the data. The FLASH instrument was subsequently employed to merge truncated reads, thereby increasing sequence quality. The identification of Amplicon Sequence Variants (ASVs) was accomplished using the Vsearch (v2.22.1.) software. This involved various stages like redundancy removal, quality evaluation, utilization of the UNOISE2 algorithm for noise reduction in sequences, chimera elimination, and alignment of reads to ASVs with a 100% similarity criterion (Rognes et al., 2016). To address underlying discrepancies in sequencing depth and ensure uniform sum of ASV sequences across samples, we applied normalization to the ASV sequence counts in the table, normalizing values to 1 to denote relative abundances. For each ASV, we selected a representative sequence using default determiner. Then, we used the latest version of QIIME2 (2022.2) (Bolyen et al., 2019) to import the rep-seqs and ASV table files. We eliminated ASVs sequences that accounted for less than 0.001% of the total across all samples. We used QIIME2’s weighted taxonomic classifiers to assign taxonomic classifications to the ASVs, and then collapsed the taxonomy from phylum to genus using the “qiime taxa collapse” command. Alpha diversity indices including Shannon, Simpson, Chao1, PD whole tree, ace, observed species were utilized to evaluate the complexity of microbial diversity in each individual sample (Liu et al., 2019). The study applied beta diversity indices to explore changes in microbial group structure. UniFrac distance metrics were employed to compare and contrast groups. Principal coordinate analysis (PCoA) and nonmetric multidimensional scaling (NMDS), two visualization methods, were used to illustrate these structural alterations (Shi et al., 2020). The analysis of similarities (ANOSIM) method was applied to determine the statistical meaning of grouping (Jiang et al., 2022). The linear discriminant analysis (LDA) and effect size calculation (LEfSe) (Erawijantari et al., 2020)were used to identify taxa that were significantly abundant in different groups. The Phylogenetic Investigation of Communities by Reconstruction of Unobserved States (PICRUSt) database and Kyoto Encyclopedia of Genes and Genomes (KEGG) were utilized for pathway enrichment analysis (Kanehisa et al., 2021). Incorporating BugBase (Wang et al., 2020), a tool created for assessing high-level characteristics of the microbiome, was used to provide a comprehensive evaluation of the composition and role of gut microbiota.

### Statistical analyses

The study conducted data analyses using two statistical software packages, R (v4.1.0) and SPSS (v26.0), focusing on both clinical and bioinformatic components. Continuous variables were reported in mean ± standard deviation format. To assess group disparates, various statistical tests were employed, covering Mann-Whitney U-tests, one-way analysis of variance or independent t-tests. For the contrast of microbial diversity and abundance in fecal samples among emICH patients and healthy individuals, the Wilcoxon rank-sum test was used. To assess differences in UniFrac distances between groups, pairwise comparisons were performed using Monte Carlo permutation test or Student’s t-test. Genus-level plots were generated using nonparametric Wilcoxon tests, with significance thresholds set at *q* < 0.1. For categorical variables, chi-square tests or Fisher’s exact tests were employed. Adjusted *p*-values were used to determine statistical significance in group comparisons, with a false discovery rate (FDR) threshold set at 0.05. Results were considered significant if the adjusted *p*-value was below 0.05.

## Results

### Clinical indicators

**Figure 2:**
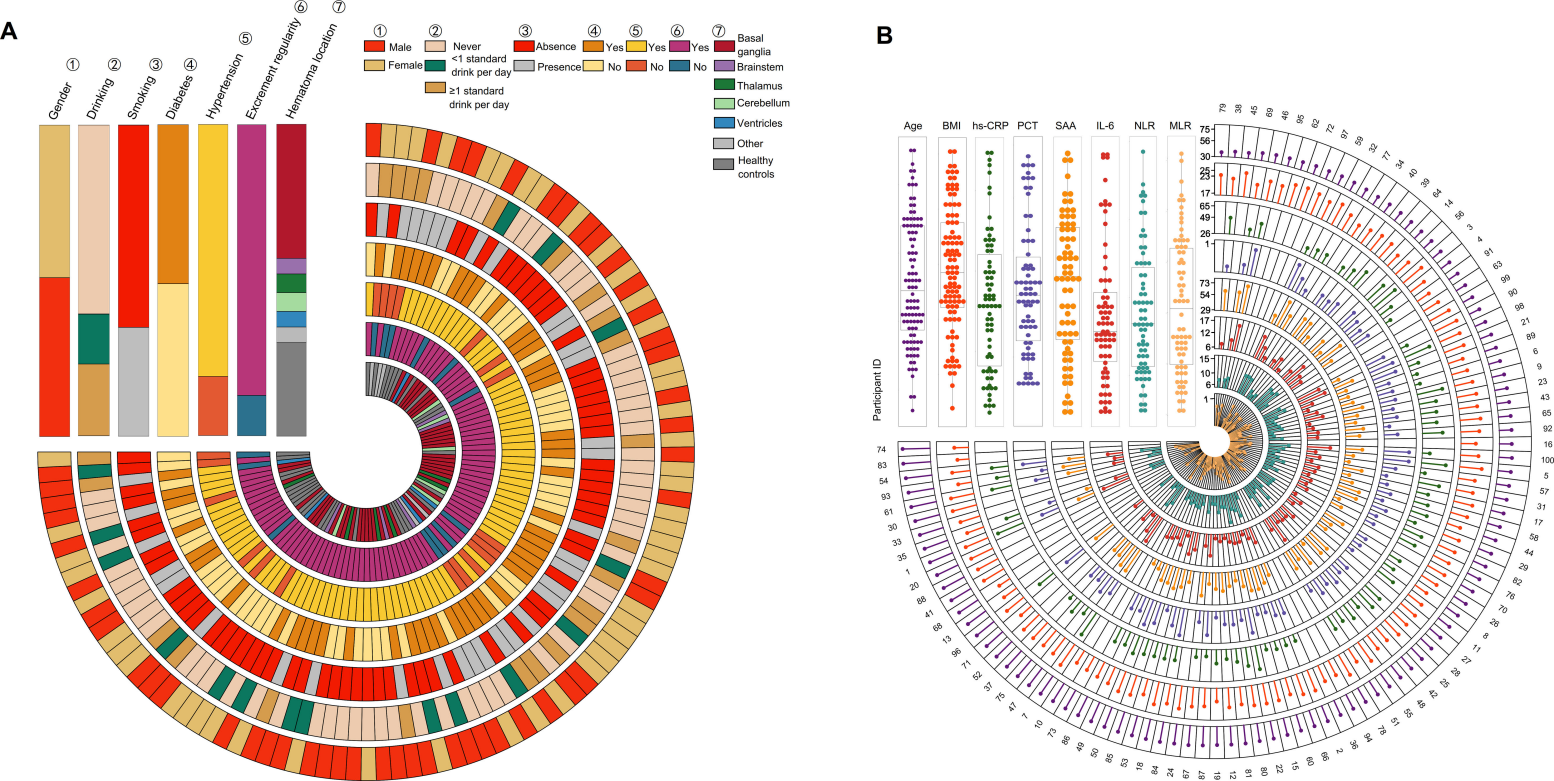
The demographics of all 100 participants.

In **Table 1**, a thorough summary of the clinical indicators is presented for each participant. Moreover, the demographics of all participants are also displayed in **Figure 2**. Notably, there were no notable differences in demographic characteristics among the groups, laying a solid basics of contrast for this study’s subsequent analysis.

**Table 1:** The baseline characteristics of all participants.

	HE	NE	HC	HC vs. ICH	HE vs. NE
	(n=30)	(n=40)	(n=30)	*p value*	*p value*
Age (Mean ± SD)	54.9 ± 14.2	53.7 ± 12.6	56.3 ± 11.8	0.639	0.747
Gender				0.896	0.448
Male	17 (56.7%)	19 (47.5%)	15 (50%)		
Female	13 (43.3%)	21 (52.5%)	15 (50%)		
BMI	23.60 ± 4.65	22.38 ± 5.51	22.92 ± 3.88	0.827	0.409
Smoking				0.253	0.414
Absence	19 (63.3%)	29 (72.5%)	17 (56.7%)		
Presence	11 (36.6%)	11 (27.5%)	13 (43.3%)		
**Drinking**				0.611	0.776
Never	19 (63.3%)	22 (55%)	20 (66.7%)		
<1 standard drink per day	4 (13.3%)	7 (17.5%)	5 (12.5%)		
≥1 standard drink per day	7 (23.3%)	11 (27.5%)	5 (12.5%)		
**Hypertension**				0.066	0.375
Positive	27 (90%)	33 (82.5%)	21 (70%)		
Negative	3 (10%)	7 (17.5%)	9 (30%)		
**Diabetes**				0.150	0.890
Negative	13(43.3%)	18 (45%)	18 (60%)		
Positive	17 (56.7%)	22 (55%)	12 (40%)		
**Excrement regularity**				0.948	0.410
Yes	25 (83.3%)	36 (90%)	26 (86.7%)		
No	5 (16.7%)	4 (10%)	4 (13.3%)		
**Hematoma location**				–	0.963
Basal ganglia	18	25	–		
Brainstem	2	3	–		
Thalamus	3	3	–		
Cerebellum	2	4	–		
Ventricles	2	3	–		
Other	3	2	–		
**Hematoma volume** (**ml, Mean ± SD**)	27.2 ± 4.9	26.8 ± 5.8	–	–	0.551
**hs-CRP** **(mg/L, Mean ± SD)**	48.63 ± 14.72	44.21 ± 12.30	–	NA	0.247
**PCT** **(ng/ml, Mean ± SD)**	0.83 ± 0.16	0.65 ± 0.11	–	NA	0.462
**SAA** **(mg/L, Mean ± SD)**	56.43 ± 17.02	52.09 ± 15.88	–	NA	0.175
**IL-6** **(pg/ml, Mean ± SD)**	12.45 ± 3.44	8.90 ± 2.73	–	NA	0.527
**NLR (Mean ± SD)**	10.66 ± 3.03	8.69 ± 2.24	–	NA	0.343
**MLR (Mean ± SD)**	0.79 ± 0.23	0.64 ± 0.18	–	NA	0.335

HC, healthy group; ICH, intracerebral hemorrhage; HE, hematoma expansion; NE, non-hematoma expansion; hs-CRP, high-sensitivity C-reactive protein; PCT, procalcitonin; SAA, serum amyloid A protein; IL-6, interleukin-6; NLR, neutrophil-to-lymphocyte ratio; MLR, monocyte-to-lymphocyte ratio; SD: Standard deviation.

### Gut microbial composition in HE, NE and HC groups

**Figure 3:**
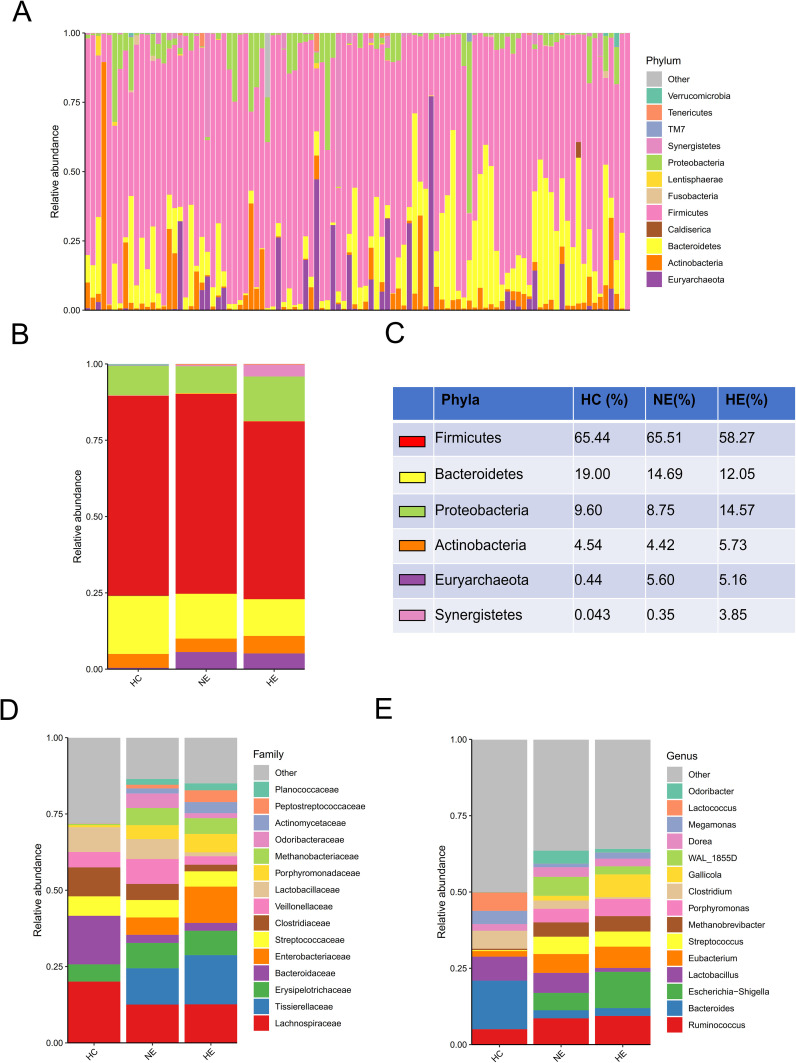
An overview of the microbial profiles identified among different groups at different taxonomic levels. The composition of microbial communities was analyzed at the phylum level for all samples **(A)** and each group **(B, C)**. The composition of microbial communities at family **(D)** and genus **(E)** levels among groups.

A comprehensive comparison of the taxonomic composition of intestinal microbiota in emICH and HC groups was conducted. The analysis revealed similar dominant phyla including *Firmicutes, Bacteroidetes, Proteobacteria, Actinobacteria*, and *Euryarchaeota* across all three groups (**Figures 3A–C**). However, there were notable differences in the composition of families and genera among the groups, indicating significant variability (**Figures 3D, E**). Additionally, detailed tables listing these bacteria (**Supplementary Table S1**) and visual representations of the top 35 bacteria based on family (**Supplementary Figure S1**) and genus (**Supplementary Figure S2**) were provided.

### Gut microbial diversity in HE

**Figure 4:**
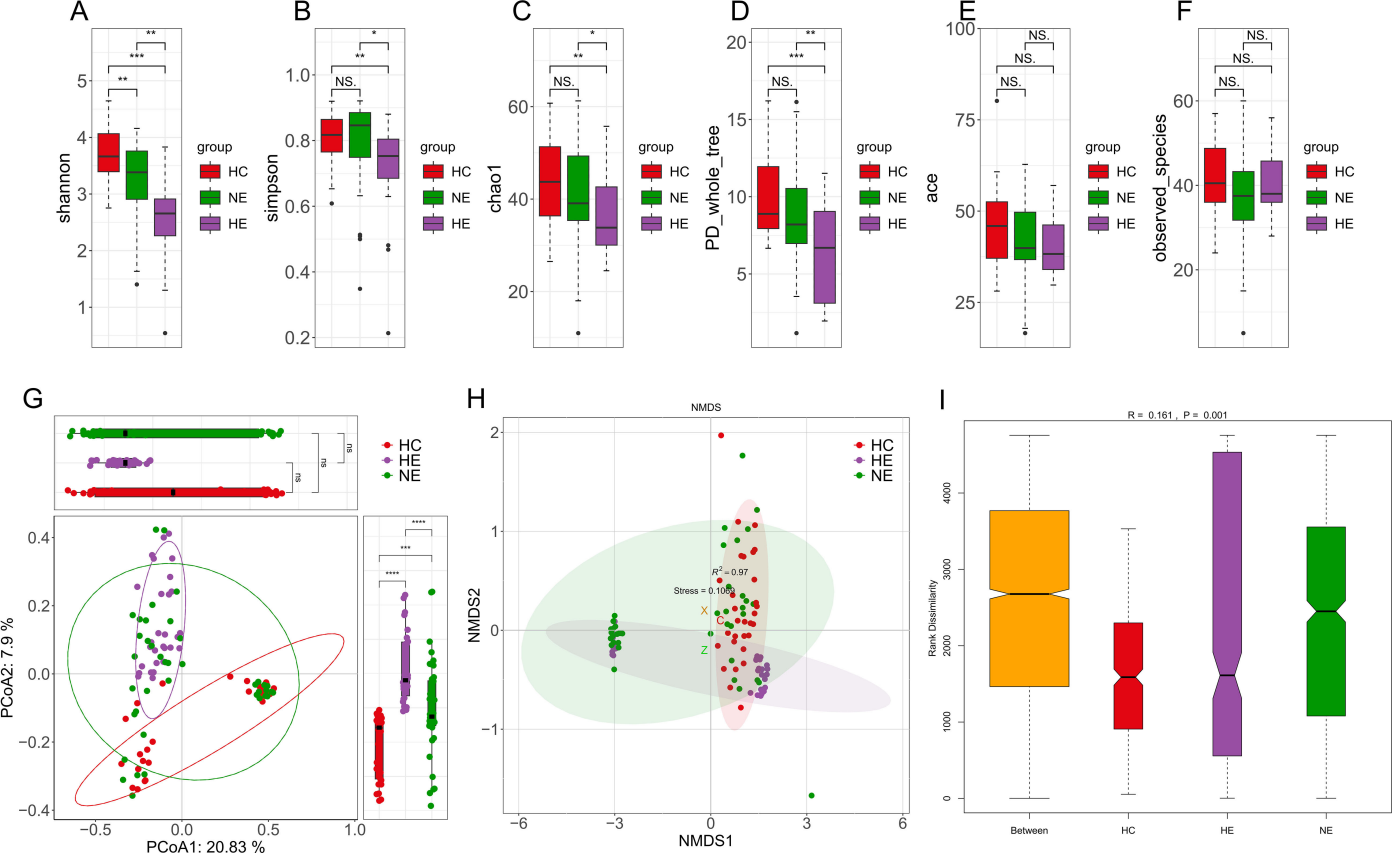
The diversity of intestinal flora. Boxplots vividly represent the α-diversity indices, including Shannon, Simpson, Chao1, PD whole tree, ace, and observed species indices **(A-F)**. The Principal Coordinate Analysis, based on unweighted UniFrac distances, visually maps the microbial landscape **(G)**. Each sample is represented by a spot on this plot, with distinct colors illustrating the separation between groups. The axes of this plot denote the primary and secondary principal coordinates, which capture the highest variance within the microbial communities. Non-metric Multidimensional Scaling (NMDS) employed Bray-Curtis distances to further describe variations in microbial composition **(H)**. Samples exhibiting high similarity in compositions of microbiome appear as closely clustered spots. The Analysis of Similarities (ANOSIM) performed on HC, HE and NE groups revealed significantly greater differences between the groups than within each group **(I)**. * 0.01 < p < 0.05; ** p < 0.01; *** p < 0.001; **** p < 0.0001. NS, No Significance.

Particularly noteworthy was the substantial variation in alpha diversity observed in the gut microbial compositions of the HE subjects when compared to both the healthy controls and the NE subjects, as demonstrated by various indices such as Shannon, Simpson, Chao1, and PD_whole_tree (**Figures 4A–D**). Conversely, no substantial differences were observed among the three groups with respect to the ace index and observed species (**Figures 4E, F**). Discriminant clustering in PCoA revealed that the intestinal flora of the HE group were obviously distinct from both the HC and NE groups (PCoA2: 7.9%, HE vs. HC, *p* < 0.0001, HE vs. NE, *p* < 0.0001, **Figure 4G**). The NMDS explain (based on Bray-Curtis distances) illustrated notable change in microbial compositions across the groups, indicating a low STRESS index of 0.1069 < 0.02 (**Figure 4H**). ANOSIM analysis highlighted their importance and meaning of distinguishing microbial ingredients, emphasizing the meaningfulness and representativeness of these groupings (*r*=0.161>0, *p*=0.001<0.05, **Figure 4I**).

### Potential microbial biomarkers for HE

**Figure 5:**
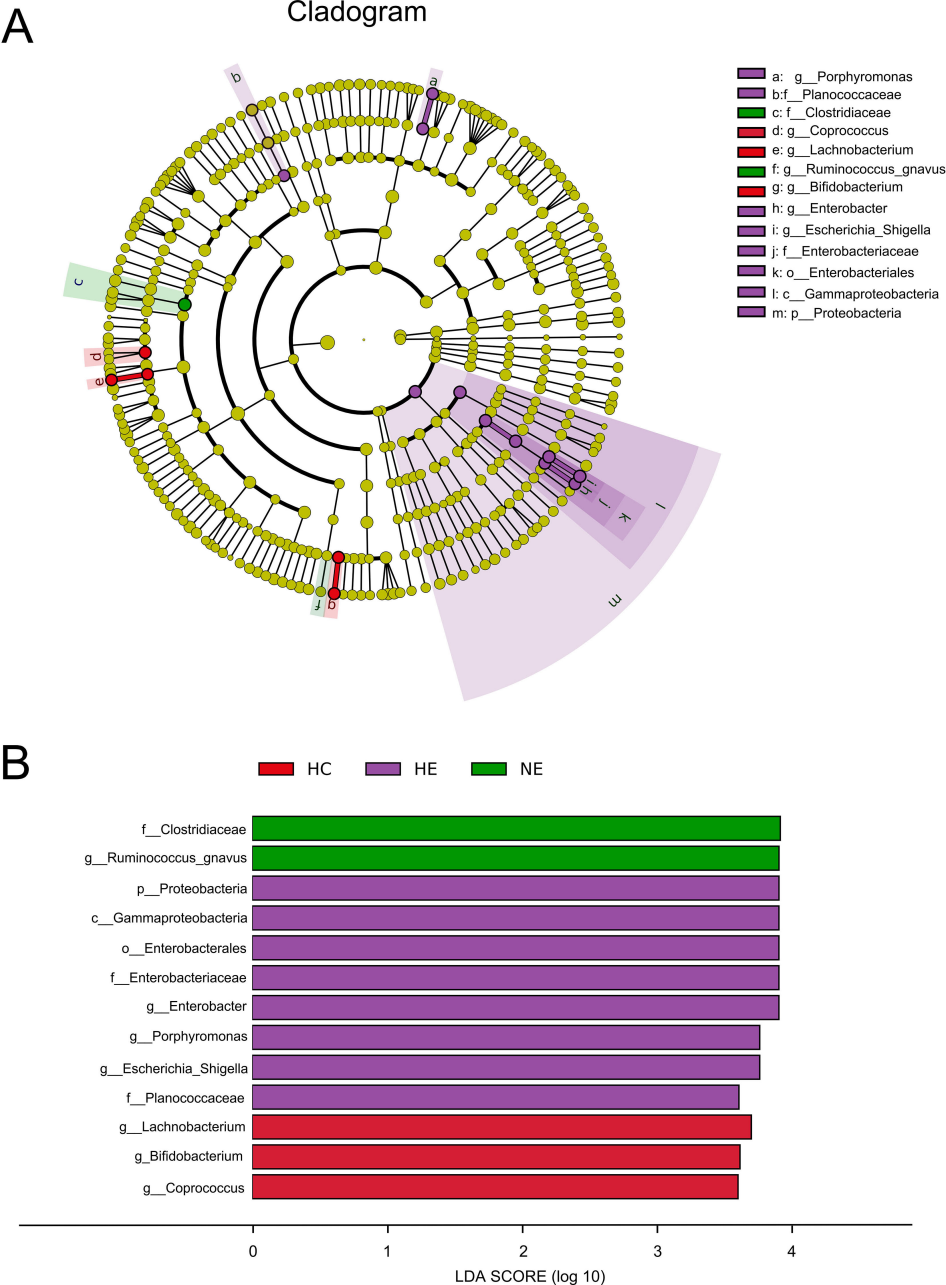
The prevalent microbial taxa in HC, HE and NE **(A)**. LDA scores highlighted the distinct taxa within the gut microbiota of different groups **(B)**. A diverse array of taxonomic ranks (including phylum and genus) is encompassed by radiations stretching from inner to outer regions. Nodes at each level signify species classifications, with disparately colored nodes denoting enrichment in their corresponding group, and yellow nodes indicating no disparate ion among groups. Taxa with LDA values greater than 4 are shown for better visualization.

Although we identified shifts in the intestinal microcolony of patients with HE and NE, but it was unable to definitively determine the main taxon solely through discriminant analysis. To address this limitation, a detailed analysis of all taxonomic ranks of gut microbiota was carried out using the LEfSe analysis tool. **Figures 5A, B** illustrated that out of the 13 microbial taxa linked to emICH, 2 were more prevalent in NE, 8 in HE, and 3 in the HC group. The emICH group, particularly the HE subgroup, exhibited harmful bacteria such as *Enterobacter, Porphyromonas*, and *Escherichia_Shigella*. Conversely, the healthy control group showed an abundance of beneficial bacteria like *Lachnobacterium* and *Bifidobacterium*.

### A panel of microbial markers for distinguishing HE from NE and HC

**Figure 6:**
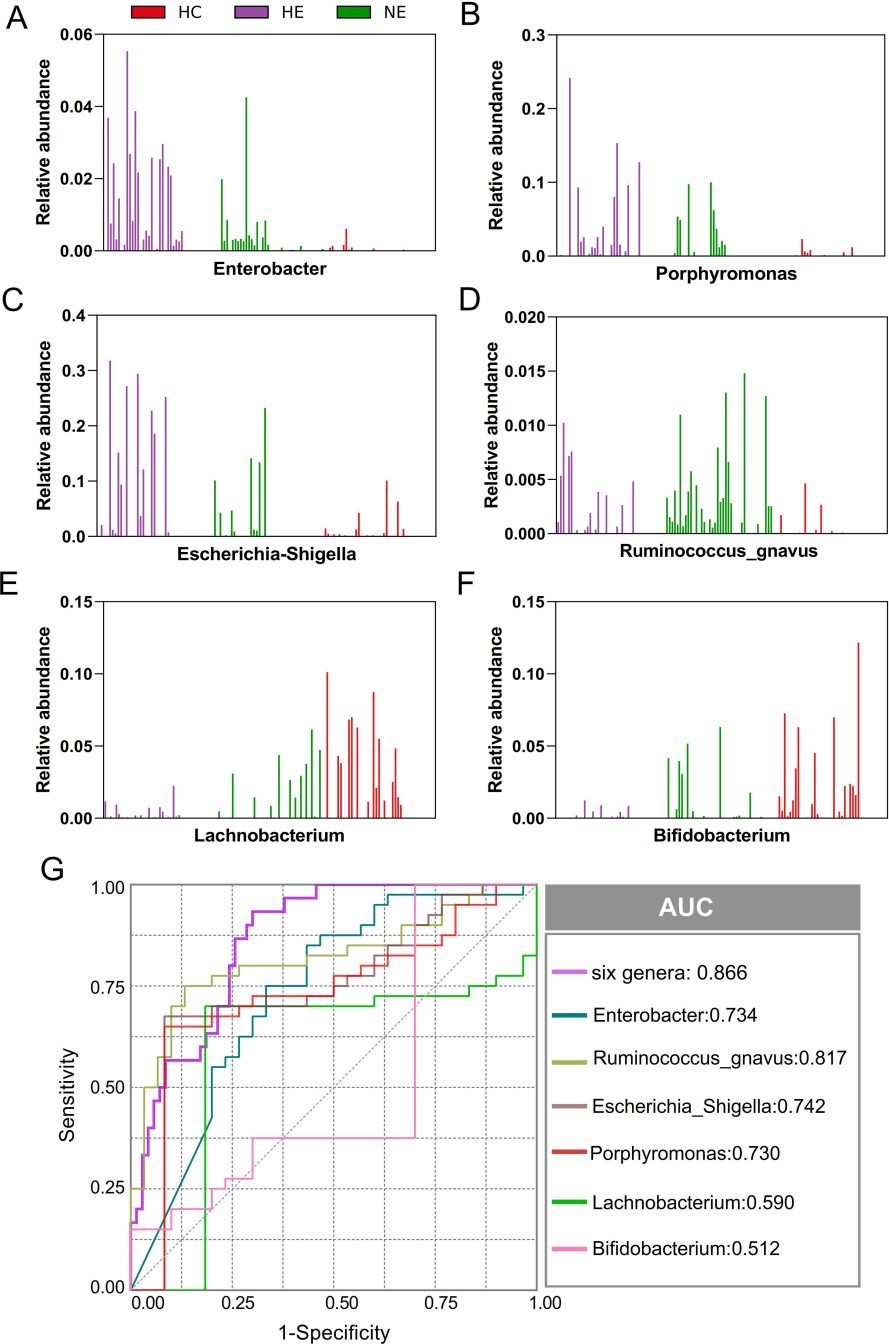
Microbial biomarker panel for predicting hematoma expansion. The abundance of six biomarkers, including *Enterobacter, Porphyromonas, Escherichia_Shigella, Ruminococcus_gnavus, Lachnobacterium*, and *Bifidobacterium*, was examined in each sample **(A-F)**. Receiver Operating Characteristic (ROC) curves were created to evaluate the ability of individual microbial genera, as well as their combined effect, to differentiate HE cases from NE and HC subjects **(G)**.

This research unveiled a variety of gut microbes linked to HE. *Enterobacter, Porphyromonas, Escherichia_Shigella, Ruminococcus_gnavus, Lachnobacterium*, and *Bifidobacterium*, were the most commonly found and consistently detected genera in emICH and HC individuals. The abundance of these genera varied significantly among individuals (**Figures 6A–F**). We conducted further analysis to determine their potential as biomarkers for predicting HE, both on their own and as a whole. The ROC curve analysis revealed that considering all six genera together substantially improved the model’s predictive accuracy, with an AUC of 0.866 (**Figure 6G**). The findings indicate a robust correlation between the identified microbial panel and the existence of HE, demonstrating the dependability and efficiency of this forecasting approach.

### Altered microbial phenotypic characteristics in HE

**Figure 7:**
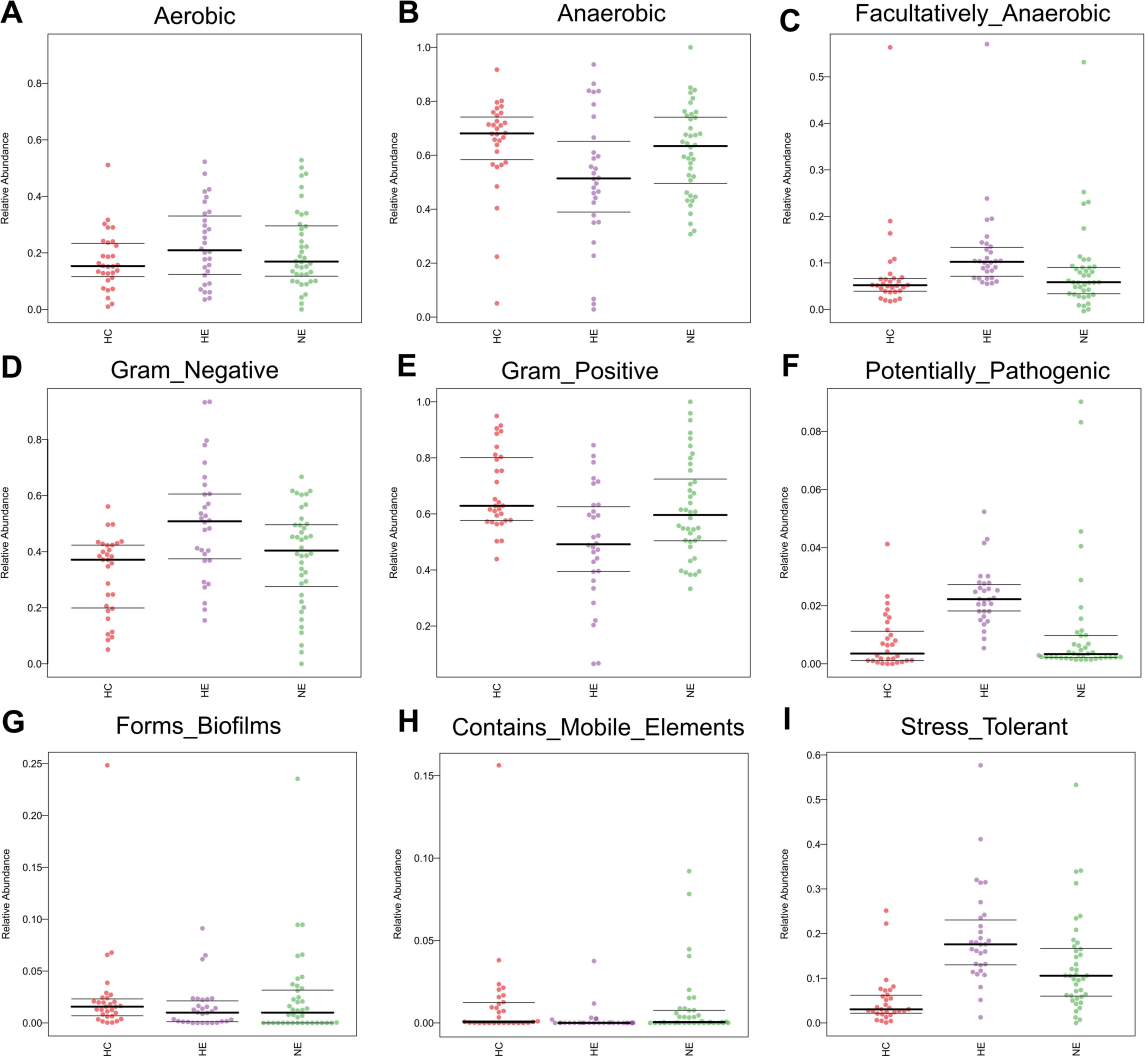
The predicted microbial phenotypic characteristics in patients with ICH, covering aerobic, anaerobic, facultatively anaerobic, gram-negative, gram-positive, potentially pathogenic, forms biofilms, contains mobile elements and stress tolerant **(A-I)**.

We performed Bugbase analysis to assess the phenotypic characteristics of bacteria. The comparison among three groups, revealed a significantly lower number of anaerobic bacteria and gram-positive bacteria in HE. The *p*-values associated with these differences were 0.014 and 0.0011, respectively. Conversely, an opposite trend was observed for the abundance of gram-negative bacteria (*p*=0.0011), opportunistic pathogens (*p*=0.0029), facultative anaerobic bacteria (*p*=0.0014), and oxidative stress tolerance (*p*=0.0015). Meanwhile, no obvious changes were discovered in biofilm formation (*p* = 0.73), mobile elements (*p*=0.13), or aerobic bacteria (*p* = 0.35) (**Figures 7A–I**).

### The correlation between gut microbial genera and blood inflammatory indicators in HE

**Figure 8:**
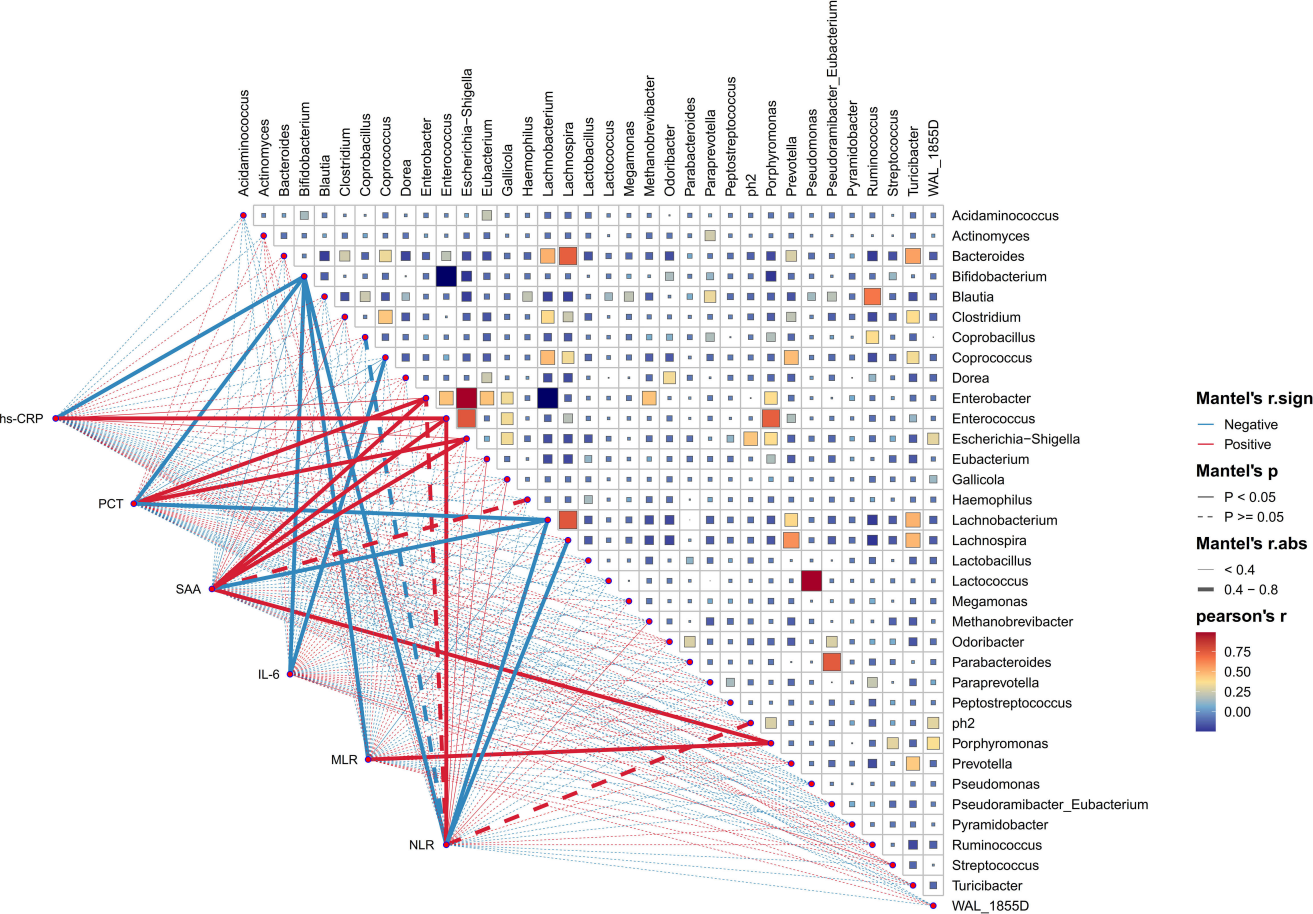
The association between diverse gut microbial genera and blood inflammation indicators. This heatmap effectively illustrates the association between 35 enriched genera in HE, evaluated through Pearson correlation analysis. The intensity of color on this heatmap corresponds to the level of correlation among these genera. Additionally, Pearson correlation analysis was utilized to investigate the relationships between these genera and the blood inflammatory indicators of HE patients. The graphical representation of these correlations includes lines, with red indicating a positive relationship and green representing the opposite. Correlation strength is indicated by the thickness of lines, with solid lines representing *p*-values lower than 0.05 and dashed lines indicating different situations. high-sensitivity C-reactive protein (hs-CRP), procalcitonin (PCT), serum amyloid A protein (SAA), interleukin-6 (IL-6), neutrophil-to-lymphocyte ratio (NLR), and monocyte-to-lymphocyte ratio (MLR).

In order to investigate into the daedal interactions between gut microbiota and blood inflammatory indicators of HE, a thorough analysis was carried out at the genus level. These underlying associations were further evaluated via the use of Pearson correlation tests between the taxa and six blood inflammatory indicators, covering hs-CRP, PCT, SAA, IL-6, NLR, and MLR. As illustrated in **Figure 8**, a complex network of relationships existed among the various bacteria populations. Notably, certain beneficial bacteria such as *Bifidobacterium* and *Lachnobacterium* (which enriched in HC) showed a strong negative correlation with these inflammatory indicators. Conversely, bacteria abundant in HE, such as *Escherichia_Shigella, Enterococcus, Enterobacter*, and *Porphyromonas*, exhibited a distinct negative correlation pattern. Meanwhile, it was not surprising that significantly abundant genera in the HC group (e.g., *Lachnobacterium* and *Bifidobacterium*) showed strong negative correlations with genera abundant in the HE group (e.g., *Enterobacter*, *Escherichia_Shigella*, and *Porphyromonas*).

### Changed microbial function profile in emICH

**Figure 9:**
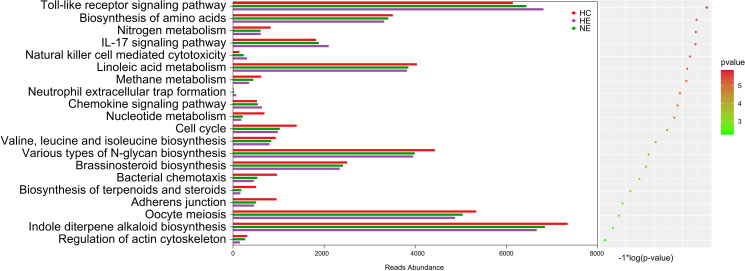
Predicted microbial functions by an overall PICRUSt analysis. This method revealed the top 20 KEGG pathway with meaning change (*p* < 0.05). KEGG, standing for the Kyoto Encyclopedia of Genes and Genomes, and PICRUSt, standing for Phylogenetic Investigation of Communities by Reconstruction of Unobserved States.

The imbalance of microbiota could trigger systemic metabolic disorders and ultimately alter the structure of gut microbiota (Fan and Pedersen, 2021). This study used PICRUSt tool to identify the 20 most different KEGG pathways among groups. The results showed that the emICH group, specifically the HE subgroup, demonstrate increased pathways associated with inflammatory responses, e.g. the Toll-ike receptor signaling pathway and the IL-17 signaling pathway. Meanwhile, it showed a declining tendency in other pathways, covering those related to metabolism and cellular processes. These findings were presented in **Figure 9**. Hence, we deduced that disturbances in gut microbiota function may contribute to an elevated risk of hematoma expansion.

## Discussion

The recent emergence of proof has brought about an increased recognition of the crucial role played by gut microbiota and its metabolites in the development and progression of various neurological illnesses (Jiang et al., 2023, 2022; Li et al., 2022), including strokes (Honarpisheh et al., 2022). Previous studies reveal that stroke has a fundamental influence on the ingredient of gut microbiota, which in turn influence the outcome of the condition. Understanding the complexities and regulation of intestinal flora might be essential for discovering novel therapeutic paths to prevent hematoma expansion in early-stage mild-to-moderate intracerebral hemorrhage (emICH).

Emerging scientific evidence has highlighted the connection between gut microbiota and ICH. A recent study by Luo et al (Luo et al., 2022)found that the presence of enriched *Enterococcus* and depleted *Prevotella* in ICH patients may significantly increase the risk of stroke-associated pneumonia. Furthermore, research by Li and colleagues (Li et al., 2023) revealed that oxymatrine could ameliorate ICH-induced white matter injury and neurological deficits by modulating gut microbiota. Nevertheless, there is still a dearth of information regarding the characteristics of peripheral immunity and intestinal flora that are linked to hematoma expansion in emICH. In this study, we examined a sample of 70 patients diagnosed with emICH, dividing them into two separate groups: the HE group (n=30) and the NE group (n=40). Each group was then compared to a control group of 30 healthy individuals to investigate the complex connections between the gut microbiome and emICH. We revealed a decrease in the diversity and abundance of microbiome in the HE group when compared to HC and NE. The distinct microbial composition within the HE group was confirmed through the application of rigorous alpha and beta diversity indices. Further analysis focused on the differences in features between the emICH and HC groups, specifically examining the gene functions of the microorganisms involved. This study aimed to develop a reliable predictive tool to accurately differentiate individuals with HE from healthy individuals and those with NE. Additionally, it sought to uncover associations between intestinal flora and blood inflammatory indicators in HE patients. The findings not only presented compelling evidence of substantial alterations in the intestinal flora of patients with emICH, but also offer insight into the intricate relationship, particularly within the context of HE.

The ingredient of gut microbiota was found to undergo disparate alterations in individuals with emICH, which is consistent with the findings reported in the relevant literature (Luo et al., 2022). The decrease in α-diversity indicates a reduction in the number of different microbial species within the groups, as well as a skewed distribution across the group. This potentially creates an environment conducive to the flourishing of the disease (Yang et al., 2019). This study has consistently demonstrated the connection between gut microbiota and emICH. The pathogen *Ruminococcus_gnavus* displayed an exceptionally high abundance in HE group. The genus *Ruminococcus gnavus* could not only cause cognitive dysfunction but also take part in the progress of oxidative stress as well as strengthen the inflammatory responses (Hall et al., 2017; Lian et al., 2021). The *Clostridiaceae*, which were enriched in the NE group, have been identified as potentially indicative of an imbalanced gut microbial environment. Furthermore, an eight-member bacterial consortium implicated in HE, consisting of typical proinflammatory pathogens like *Enterobacter*, *Escherichia_Shigella*, and *Porphyromonas*, were clearly observed. The genera *Enterobacter* and *Escherichia_Shigella* belong to the *Enterobacteriaceae* family, which is recognized for producing pathogen-associated molecular patterns (Cattaneo et al., 2017). Both of them were proved associated with neurological diseases by causing inflammation, neurotoxicity and cognitive impairment (Kitamoto et al., 2020; Streit et al., 2021). In addition, studies have demonstrated that *Porphyromonas* could also stimulate the production of pro-inflammatory mediators and enhances the aggregation of β-amyloid, a protein that has been linked to neurotoxic influence in the brain (Jungbauer et al., 2022). The study found that individuals in the HE group had a more severe gut microbiota dysbiosis, suggesting that the degree of microbial dysbiosis is associated with an increased risk of HE. Aside from examining possible pathogens, we also identified less common microbes in the HE group, including *Bifidobacterium* and *Lachnobacterium*. These microbes are known for their probiotic properties, which offer anti-inflammatory benefits primarily through the synthesis of butyric acid (Nie et al., 2021; Yi et al., 2021). Furthermore, *Bifidobacterium* has been shown to alleviate inflammation associated with macrophages both locally and systemically in a manner that relies on TLR4/MyD88 and NLRP3/Caspase1 (Ehrlich et al., 2020; Li et al., 2022). Probiotics, on the other hand, have demonstrated the ability to consistently scavenge high reactive oxygen species and reduce inflammatory factors (Cao et al., 2023). Hence, the decreased concentration of beneficial bacteria may be viewed as a potential risk factor for HE. The results indicated a bidirectional connection between the disrupted gut microenvironment and the impacted central nervous system in emICH, and suggested that the gut microbiota is expected to be used as a predictive instrument for HE.

In order to fully comprehend the emICH (covering HE and NE groups) gut microbiome, it is important to investigate into the dynamic network of interactions among disparate microorganisms. We posited that the microbiome’s antagonistic and synergistic connections, rather than the mere existence or non-existence of individual bacteria could be vital in the progress of hematoma expansion. The lipopolysaccharide of gram-negative bacteria, which was notably enriched in the HE group (Maldonado et al., 2016), has been demonstrated to potentially irritate the growth of facultative anaerobes through its role as a source of energy (Yoo et al., 2020). Based on the observed correlation, we can reasonably assume that there is an imbalance in the oxygen concentration within the gut environment. This might explain the increase in the presence of *Enterobacteriaceae*, while the population of strictly anaerobic gut bacteria has decreased (Yoo et al., 2020). The elevated level of *Enterobacteriaceae* might subsequently irritate neutrophil transepithelial migration, leading to the diminishment of beneficial bacteria e.g. *Bifidobacterium* and *Lachnobacterium* (Li et al., 2023). During this process, the decline in probiotic levels could limit their effectiveness in restricting *Enterobacteriaceae* by reducing intestinal pH (Behnsen, 2017; Yoo et al., 2020). Eventually, a harmful cycle could form. This elaborate cascade underscored the intricate dynamics within the intestinal flora and their possible effects on the early expansion of hematoma in ICH.

Furthermore, correlation analysis between dominating genera and blood inflammatory parameters within HE group was carried out to better understand microbial contribution to early expansion of hematoma. The bacteria, specifically *Enterobacter*, *Escherichia_Shigella*, and *Porphyromonas*, that were abundant in the HE group displayed favorable correlations with inflammatory indicators, consistent with results reported in prior research studies (Posawetz et al., 2021). Multiple studies have showed that ICH is frequently accompanied by dysbiosis of the gut microbiota and systemic inflammatory response, with the degree of inflammation serving as a predictor for hematoma expansion. Even with these well-known facts, the specific connections among hematoma expansion, inflammatory response and disturbed microbiota, are not yet well understood. Significantly, the HE group was abundant in gram-negative bacteria capable of secreting lipopolysaccharide (LPS) (Di Lorenzo et al., 2022). Research has identified various mechanisms by which LPS could induce a systemic inflammatory response, e.g. activation of the TLR4 pathway and impairment of colonic epithelial permeability (Gu et al., 2022; Xu et al., 2021). Therefore, we hypothesized that the disturbed intestinal flora could exacerbate the inflammatory response and increase the risk of hematoma expansion.

**Figure 10:**
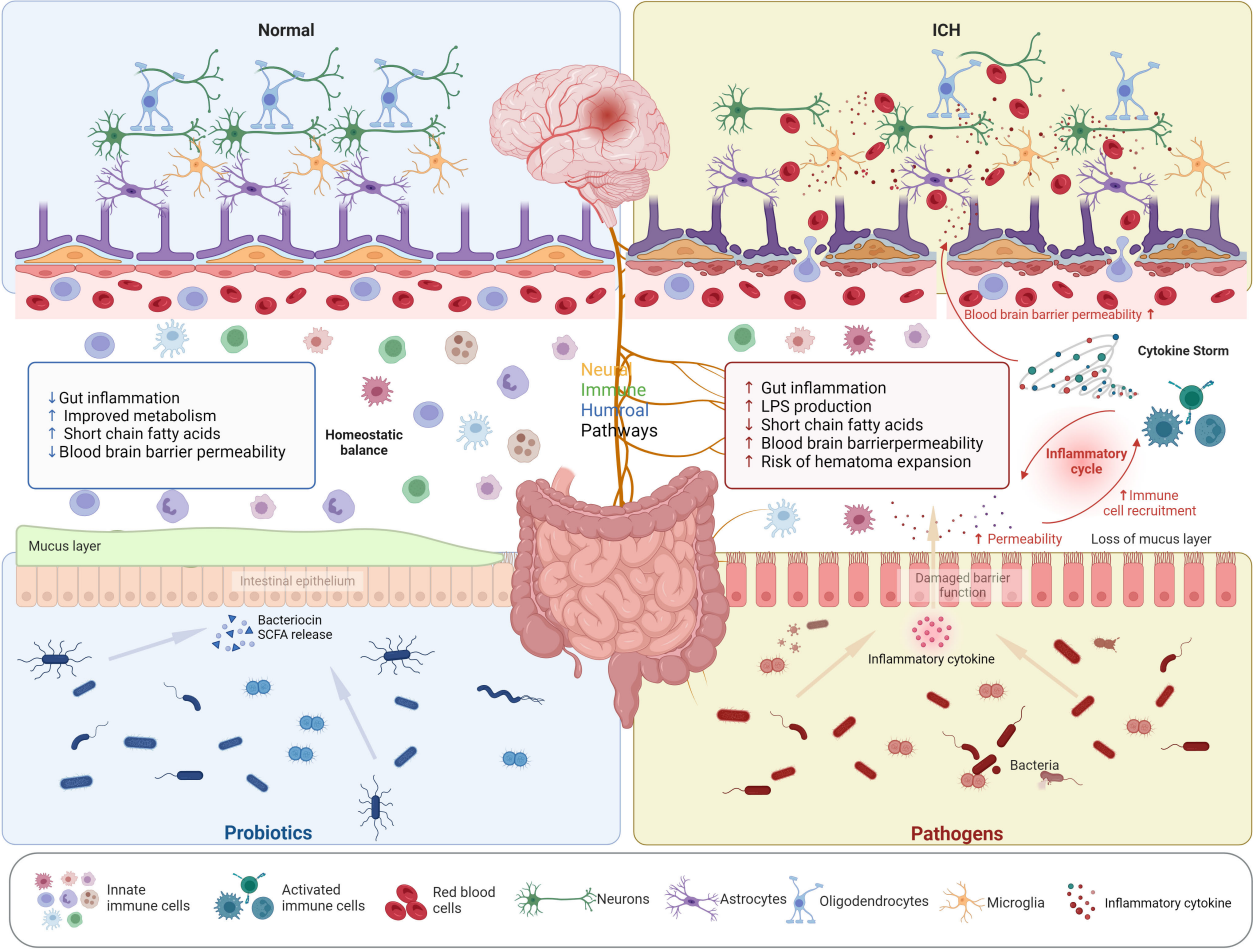
Interaction between intestinal dysbiosis and hematoma expansion in intracerebral hemorrhage. The composition of enriched intestinal flora in the HC, HE, and NE group is listed in **Supplementary Table S1**.

Further analysis was conducted using KEGG analysis to validate the hypothesis. The most surprising finding was the significant increase in inflammatory responses activity within HE group, particularly in the TLR (Toll-like receptor) signaling pathway and the IL-17 signaling pathway. As previously documented, both IL-17 and TLR signaling pathways are classic inflammatory pathways implicated in various diseases (McGeachy et al., 2019; Wang et al., 2004), including stroke (Swardfager et al., 2013). Furthermore, decreased abundance were also revealed in the pathway connected to metabolism and cellular processes in HE. It may be caused by the diminished microbial ecosystem in the HE group. In summary, the complex interrelations between HE and the intestinal flora are not yet fully understood. Concurrently, disturbances at the microbiome-metabolome junction might contribute to the onset and progression of HE. It seems plausible to suggest that this condition might be initiated by pro-inflammatory toxins or metabolites that influence immune and inflammatory responses. **Figure 10** may illustrate this assumption in a more intuitive manner. Hence, targeting these specific pathways may offer potential strategies for preventing hematoma expansion.

This study represents the initial comprehensive examination of significant changes in microbial composition and function in patients with HE. Particularly important is a significant decrease in beneficial microorganisms such as *Bifidobacterium* and *Lachnobacterium*, along with an increase in gram-negative pathogens that cause inflammation, and disruptions in inflammation-related pathways. These findings underscore the crucial predictive and preventive role of intestinal flora in hematoma expansion after ICH. Meanwhile, we propose the idea of supplementing ICH patients with a probiotic mixture upon their admission to the hospital, with the aim of preventively reducing the risk of hematoma expansion. Moreover, investigating animal models could provide valuable understanding of new therapeutic strategies for HE, including targeted modification of intestinal flora through particular drugs or fecal microbiota transplantation (which could be used to explore the underlying causal relationship between hematoma expansion and intestinal flora dysbiosis).

This research acknowledges a number of inherent limitations which suggests that further research is required. At first, the cross-sectional design means that it is not possible to establish a definitive causal relationship between the disturbed microbiota and HE. Additionally, the limited sample size may hinder the widespread applicability of the results. Thirdly, specific subtypes of ICH were not classified (basal ganglia hemorrhage, cerebellar hemorrhage, brainstem hemorrhage, etc.), ignoring the fact that the flora may exist differences between different subtypes. Moreover, due to the inherent complexity of intestinal flora, the elimination of all latent variables and biases can be tremendously challenging. Another significant constraint is the absence of well-structured animal studies that examine fundamental mechanisms. In order to overcome these obstacles, future studies should adopt more advanced experimental methodologies.

## Conclusions

A notable decrease in microbial diversity was observed in HE patients. Furthermore, we identified specific microbial markers associated with HE, covering *Escherichia_Shigella*, *Enterobacter*, and *Porphyromonas*. Interestingly, several bacteria abundant in HE were also strongly correlated with inflammatory blood indicators, suggesting a underlying link between these elements. By analyzing KEGG pathways, it was determined that the main influence of disrupted gut microbiota on HE is mediated by inflammatory reactions, specifically involving the Toll-like receptor signaling pathway. As a result, we suggest that investigating a new treatment target related to microbiota control shows potential for predicting and preventing HE, and should be further explored.

## Data Availability

The data presented in the study are deposited in the NCBI repository, accession number PRJNA1212289.

## Supplementary material

Supplementary Figure 1 presents a heatmap visualization of the 35 most abundant taxa, categorized at the family level across all specimens.

Supplementary Figure 2 presents a heatmap displaying the 35 most abundant taxa at the genus level across all samples.
