# Supplementary figures and images for: Exploring the associations of gut microbiota with inflammatory and the early hematoma expansion in intracerebral hemorrhage: from change to potential therapeutic objectives
Source: Front Cell Infect Microbiol. 2025 Feb 3;15:1462562. doi: 10.3389/fcimb.2025.1462562 (PMC11830820; doi:10.3389/fcimb.2025.1462562)

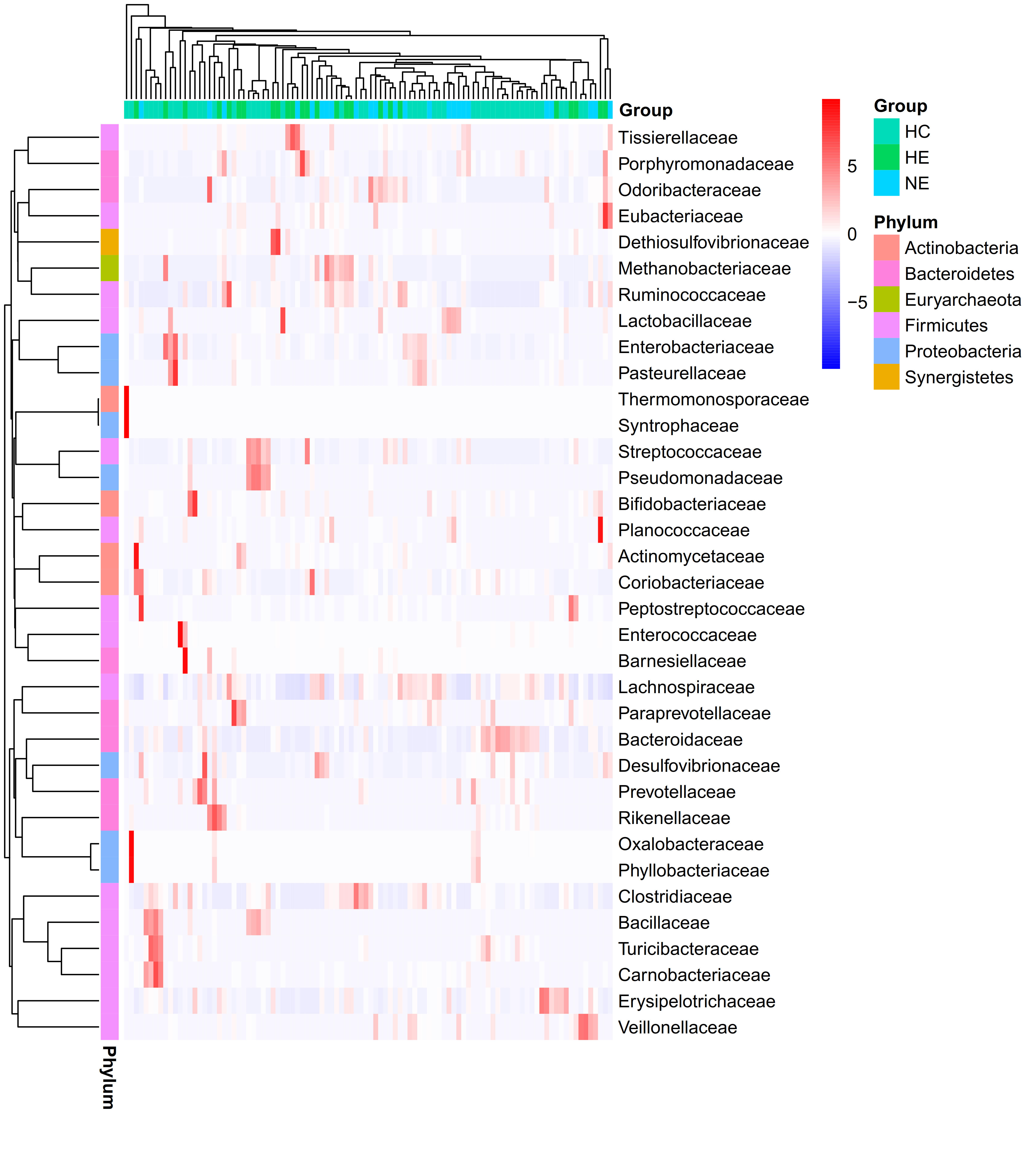

Supplement: Supplementary Figure 1 — presents a heatmap visualization of the 35 most abundant taxa, categorized at the family level across all specimens. [file Image1.jpg]

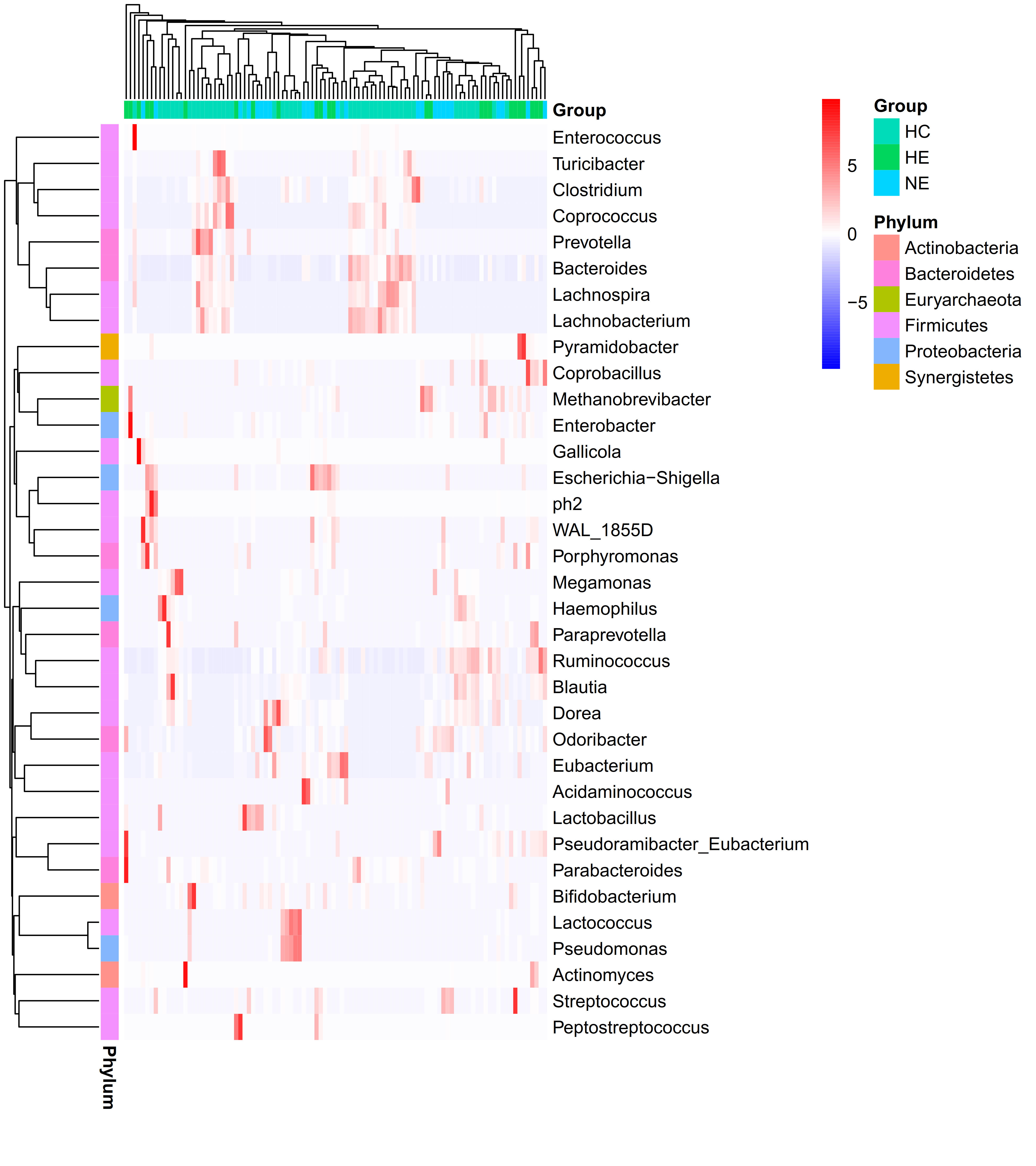

Supplement: Supplementary Figure 2 — presents a heatmap displaying the 35 most abundant taxa at the genus level across all samples. [file Image2.jpg]
