# Supplementary material for: Exploring the associations of gut microbiota with inflammatory and the early hematoma expansion in intracerebral hemorrhage: from change to potential therapeutic objectives
Source: Front Cell Infect Microbiol. 2025 Feb 3;15:1462562. doi: 10.3389/fcimb.2025.1462562 (PMC11830820; doi:10.3389/fcimb.2025.1462562)
Supplement: Supplementary file 3 [file Table1.docx]

**Supplementary Table S1. The relative abundance of** **[dominan](javascript:;)t gut microbiota among HC, NE and HE groups.**

|  | HC | NE | HE |
| --- | --- | --- | --- |
| f__Lachnospiraceae | 0.200720314 | 0.125470037 | 0.126218949 |
| f__Tissierellaceae | 0.000211903 | 0.119009364 | 0.161183711 |
| f__Erysipelotrichaceae | 0.056302282 | 0.082911004 | 0.079965396 |
| f__Bacteroidaceae | 0.15906734 | 0.026576465 | 0.025555349 |
| f__Enterobacteriaceae | 8.00E-06 | 0.056942197 | 0.119035553 |
| f__Streptococcaceae | 0.063818848 | 0.057160037 | 0.050179362 |
| f__Clostridiaceae | 0.094824685 | 0.052728716 | 0.021583599 |
| f__Veillonellaceae | 0.050352999 | 0.081443451 | 0.02777719 |
| f__Lactobacillaceae | 0.081282871 | 0.065340498 | 0.012717117 |
| f__Porphyromonadaceae | 0.006820884 | 0.0458037 | 0.060378113 |
| f__Methanobacteriaceae | 0.004358009 | 0.055979116 | 0.05158263 |
| f__Odoribacteraceae | 0.000955564 | 0.047689735 | 0.016246169 |
| f__Actinomycetaceae | 7.20E-05 | 0.016647553 | 0.036363854 |
| f__Peptostreptococcaceae | 0.000387823 | 0.012004124 | 0.038848807 |
| f__Planococcaceae | 8.80E-05 | 0.018911941 | 0.022510758 |
| g__Ruminococcus | 0.050468947 | 0.086006623 | 0.093885314 |
| g__Bacteroides | 0.15906734 | 0.026576465 | 0.025555349 |
| g__Escherichia-Shigella | 8.00E-06 | 0.056942197 | 0.119035553 |
| g__Lactobacillus | 0.078568111 | 0.065340498 | 0.012717117 |
| g__Eubacterium | 0.018203685 | 0.061809199 | 0.069549472 |
| g__Streptococcus | 0.003526389 | 0.056930732 | 0.04979931 |
| g__Methanobrevibacter | 0.004358009 | 0.046858504 | 0.050392358 |
| g__Porphyromonas | 0 | 0.0444508 | 0.056510773 |
| g__Clostridium | 0.058773153 | 0.027029343 | 0.005458546 |
| g__Gallicola | 0 | 0.015684471 | 0.074778316 |
| g__WAL_1855D | 0 | 0.062107295 | 0.026449097 |
| g__Dorea | 0.021937979 | 0.031374675 | 0.025112651 |
| g__Megamonas | 0.043348199 | 0.011740423 | 0.019850396 |
| g__Lactococcus | 0.060292459 | 0.000229305 | 0.000380052 |
| g__Odoribacter | 0.000911584 | 0.042409984 | 0.011689724 |

Abbreviations: HC, healthy control; HE, hematoma expansion; NE, non-hematoma expansion; p, phylum; f, family; g, genus.
